# Characterization of the *F* Locus Responsible for Floral Anthocyanin Production in Potato

**DOI:** 10.1534/g3.120.401684

**Published:** 2020-08-27

**Authors:** F. Parker E. Laimbeer, Bastiaan O.R. Bargmann, Sarah H. Holt, Trenton Pratt, Brenda Peterson, Andreas G. Doulis, C. Robin Buell, Richard E. Veilleux

**Affiliations:** * School of Plant and Environmental Sciences, Virginia Tech, Blacksburg VA 24061; † Department of Biology, University of North Carolina, Chapel Hill NC 27599; ‡ Hellenic Agricultural Organization DEMETER (ex. NAGREF), Heraklion, Greece; § Department of Plant Biology, Michigan State University, East Lansing MI 48824

**Keywords:** *Solanum tuberosum*, bulk segregant analysis, copy number variation, transgenic complementation, monoploid

## Abstract

Anthocyanins are pigmented secondary metabolites produced via the flavonoid biosynthetic pathway and play important roles in plant stress responses, pollinator attraction, and consumer preference. Using RNA-sequencing analysis of a cross between diploid potato (*Solanum tuberosum* L.) lines segregating for flower color, we identified a homolog of the *ANTHOCYANIN 2* (*AN2*) gene family that encodes a MYB transcription factor, herein termed *StFlAN2*, as the regulator of anthocyanin production in potato corollas. Transgenic introduction of *StFlAN2* in white-flowered homozygous doubled-monoploid plants resulted in a recovery of purple flowers. RNA-sequencing revealed the specific anthocyanin biosynthetic genes activated by *StFlAN2* as well as expression differences in genes within pathways involved in fruit ripening, senescence, and primary metabolism. Closer examination of the locus using genomic sequence analysis revealed a duplication in the *StFlAN2* locus closely associated with gene expression that is likely attributable to nearby genetic elements. Taken together, this research provides insight into the regulation of anthocyanin biosynthesis in potato while also highlighting how the dynamic nature of the *StFlAN2* locus may affect expression.

Anthocyanins are a group of secondary plant metabolites which provide various benefits to plants, such as pollinator attraction and resistance to biotic and abiotic stressors including cold, ultraviolet light, and oxidative stress (Chalker‐Scott 1999; Merzlyak and Chivkunova 2000; Steyn *et al.* 2002; Schulz *et al.* 2016). Agronomically, anthocyanins play to consumer preference in fruit and vegetables while also providing a source of dietary antioxidants that confer health benefits (de Pascual-Teresa *et al.* 2010; Alvarez-Suarez *et al.* 2014; Khoo *et al.* 2017). In potato, anthocyanins can accumulate in a wide range of tissues including leaves, stems, flowers, and tubers. Thus, study of anthocyanins in potato is important for their role in consumer health and appeal as well as the potential for stress mitigation which could affect agronomic traits such as yield and storage quality.

Anthocyanins are a broad class of flavonoids which vary in the state and type of glycosylation. Anthocyanidin precursors are produced through the phenylpropanoid pathway, beginning with the catabolism of phenylalanine and a chalcone intermediary (Tanaka *et al.* 2008; Petroni and Tonelli 2011). The remaining biosynthetic steps and the model of their regulation, are summarized in Figure 1. Briefly, chalcone is converted to dihydrokaempferol by a series of steps mediated by chalcone isomerase (*CHI*) and flavonoid 3-hydroxylase (*F3H*). Next, modifications by flavonoid 3′ hydroxylase (*F3′H*) and flavonoid 3′-5′ hydroxylase (*F3′5’H*) can alter the specific flavonoid precursor, determining the type of anthocyanin synthesized. Dihydroflavonol 4-reductase (DFR) catalyzes dihydroflavanones to leucoanthocyanidins, which then are converted to anthocyanidin by anthocyanindin synthase (ANS) (Wang *et al.* 2013). ANS and various glucosyl transferases complete the pathway, giving an anthocyanin its specific identity. While the structural enzymes are well conserved, their regulation differs by clade. In solanaceous species, the early biosynthetic genes leading to production of flavonoids are regulated by R2R3 MYBs, which are often tissue-specific (Jung *et al.* 2009). The late biosynthetic genes, which affect the modification of the flavonoids and anthocyanins, are regulated by a heterocomplex of MYB, basic helix loop helix (bHLH), and WD40 transcription factors (de Vetten *et al.* 1997; Quattrocchio *et al.* 1999; Spelt *et al.* 2000; de Pascual-Teresa *et al.* 2010; Feller *et al.* 2011).

**Figure 1:**
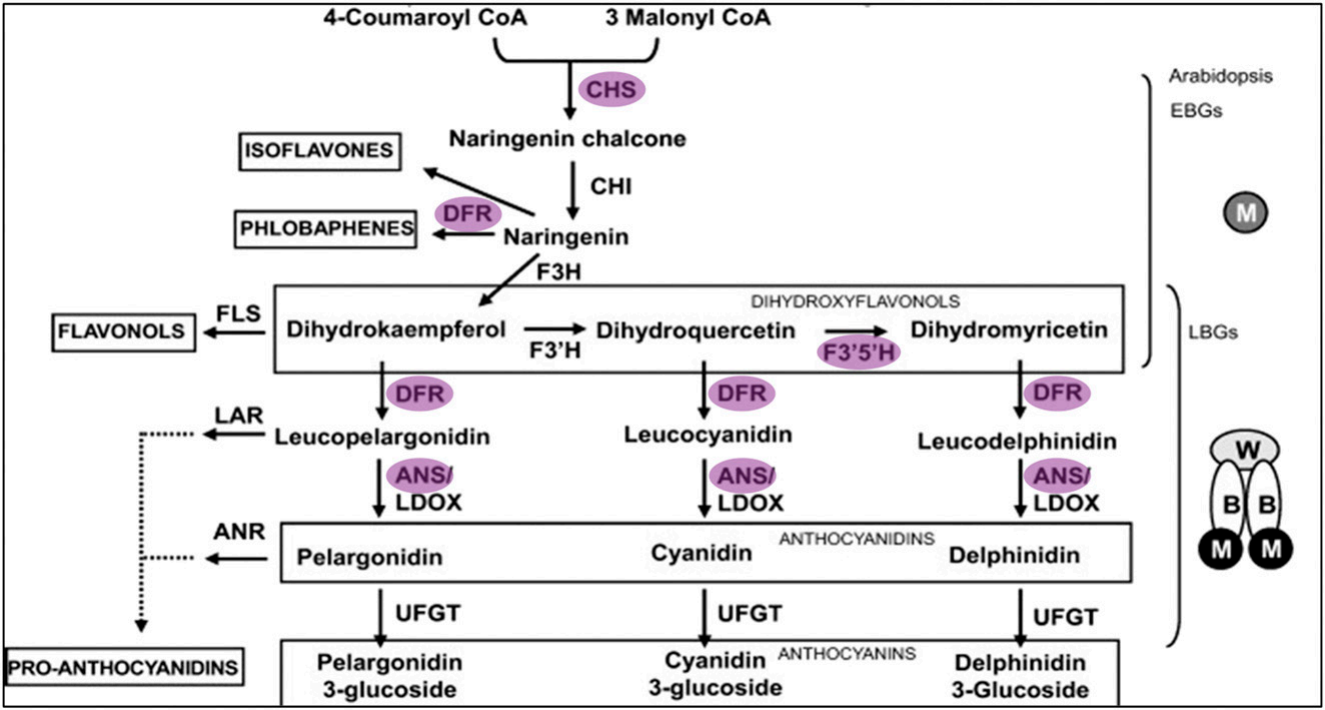
A simplified scheme of anthocyanin synthesis and regulation adapted from Petroni & Tonelli, 2011, with permission from Elsevier. Enzyme abbreviations are as follows: Chalcone synthase (CHS), chalcone isomerase (CHI), flavonol synthase (FLS), flavonoid 3 hydroxylase (F3H), flavonoid 3′ hydroxylase (F3′H), flavonoid 3′5’ hydroxylase (F3′5’H), dihydroflavonol reductase (DFR) anthocyanidin synthase/leucoanthocyanin oxidase (ANS/LDOX), anthocyanidin reductase (ANR), UDP- flavonol glucosyltransferase (UFGT). Transcription factor abbreviations are as follows: MYB transcription factor (M), WD 40 (W), basic helix-loop-helix (B). EBGs and LBGs refer to early and late biosynthetic genes respectively. Abbreviations highlighted in purple were significantly up-regulated in purple flowered DRH bulks.

In petunia (*Petunia hybrida*), which is often used as a model organism for the study of anthocyanin production, expression of the early biosynthetic genes is initiated in flowers by a MYB transcription factor denoted as *PhAN2. PhAN2* also controls expression of the bHLH transcription factor, *PhAN1*, which regulates the late biosynthetic genes (Quattrocchio *et al.* 1999; Spelt *et al.* 2000). In potato tubers, separate loci have been proposed to control tuber skin pigmentation: *D*, *P* and *R*. The *D* locus is required for any skin pigmentation, while *P* and *R* control purple or red skin color (Dodds and Long 1955). All three loci have been mapped and cloned. Subsequent analysis has shown that *D* encodes a MYB transcription factor on chromosome 10 with high sequence identity to *PhAN2*, currently called *StAN1* (Payyavula *et al.* 2013; D’Amelia *et al.* 2014). There has been discrepancy in the naming of *StAN1* as it was concurrently characterized and named both *StAN1* and *StAN2*; in current literation and herein *StAN1* will refer to the potato homolog of *PhAN2* (Jung *et al.* 2009; Payyavula *et al.* 2013; D’Amelia *et al.* 2014; Liu *et al.* 2016). *P* and *R* encode the biosynthetic genes, *F3′5’H* on chromosome 11 and *DFR* on chromosome 2, respectively (De Jong *et al.* 2004; Jung *et al.* 2005; Zhang *et al.* 2009a). However, the control of tuber flesh pigmentation is not as clear. A study by Zhang *et al.* (2009b) showed that a homolog of the bHLH transcription factor *PhAN1* [initially called *StAN1* but since renamed *StbHLH1*; (Payyavula *et al.* 2013; D’Amelia *et al.* 2014)] mapped to chromosome 9 and plays a significant role in, but is not wholly responsible for, anthocyanin accumulation in tuber flesh. Later studies showed that there is substantial allelic diversity in the C-terminal region, specifically the R-repeat, of the R2R3 MYBs, such as *StAN1*, and in the bHLH transcription factors (*StbHLH1* and *StJAF13*) that act as co-regulators, resulting in a variety of tuber pigmentations across genotypes (Liu *et al.* 2016; Strygina *et al.* 2019).

Similar to tubers, three potato flower loci have been described: *F*, *D*, and *P* (van Eck *et al.* 1993). The *F* locus is required for any floral pigmentation, whereas *D* and *P* control color shade. The floral *D* locus, responsible for the biosynthesis of red anthocyanins, maps to chromosome 2 and appears to correspond to the tuber *R* locus (*DFR*). The floral *P* locus is responsible for purple anthocyanin accumulation, localizes to chromosome 11, and appears to correspond to the tuber *P* locus (*F3′5’H*). The same study used restriction fragment length polymorphism analysis to map the *F* locus to chromosome 10, nearby the tuber *D* locus. Since purple tuber skins are observed in plants with white flowers and *vice versa*, it is plausible that there are multiple homologs of *PhAN2* (which may have arisen via duplication on chromosome 10) that independently dictate pigment accumulation in different tissues.

As the genetic mechanism of anthocyanin production in potato flowers has received considerably less investigation than anthocyanin production in tubers, we set out to investigate the floral *F* locus. Using a segregating diploid population in conjunction with inbred and homozygous individuals derived from that population, we identified a separate *PhAN2* homolog that underlies the floral *F* locus. An RNA-sequencing (RNA-seq) approach revealed the regulatory cascade caused by this homolog while analysis of the locus itself denoted duplications that may underlie differences in gene expression and floral phenotypes.

## Materials And Methods

### Plant material used in this study

The population segregating for purple or white flower color was generated as described by Peterson *et al.* (2016). Briefly, it is derived from a diploid cross between a white-flowered doubled monoploid, DM 1-3 516 R44, and a heterozygous purple-flowered individual, RH89-039-16 (Potato Genome Sequencing Consortium 2011). In this study, the F_1_ population (DRH), comprised of 95 individuals, was screened for flower color, with the white-flowered plants designated DRH_W_ and the purple-flowered plants designated DRH_P_. Plants were grown in growth chambers with a 16 h photoperiod, 250 μE m^−2^ s^−1^, 22° days, and 18° nights. An inbred (S_5_) individual derived from DRH and fixed for purple flowers, designated DRH_P_ 28-5, was obtained through successive rounds of self-pollinations. To generate monoploids from F_1_ individuals, we conducted anther culture on immature flower buds of numerous DRH plants (Paz and Veilleux 1999), with regenerated plants screened by flow cytometry to identify monoploid individuals according to Teparkum and Veilleux (1998). In addition, nine other monoploid potato clones available from a previous study (Hardigan *et al.* 2016) were characterized for flower color and sequence. The entirety of germplasm used is presented in Supplementary Figure 1 and Supplementary Table 1.

### Genotype analysis

SNP-chip genotype data from Peterson *et al.* (2016) were analyzed using the purple/white phenotype of the F_1_ segregating population. Briefly, the Infinium platform (Illumina, Inc.; 8303 SNP array for potato (Felcher *et al.* 2012)) was used to genotype 95 DRH F_1_ plants with known flower color (44 DRH_W_ and 51 DRH_P_). Genotyping calls were made using an Illumina iScan reader with the Infinium HD Assay Ultra and allele calls using GenomeStudio (Illumina, Inc.).

### Gene expression profiling

Samples were collected by combining corollas from ten DRH_P_ or ten DRH_W_ individuals from the DRH F_1_ segregating population; two samples of ten corollas were collected for each color. RNA was extracted using a hybrid trizol/Qiagen RNeasy mini kit extraction protocol (https://microarray.adelaide.edu.au/protocols/), including a DNase treatment (Ambion Catalog # AM 1906) and followed by quality assessment on a Bioanalyzer (Agilent). Illumina RNA-seq libraries were constructed and sequenced on Hi-Seq 2500 platform to obtain 100 nt paired-end reads. All read quality control and subsequent analysis were performed using the CLC Genomics Workbench 7.5. Reads were trimmed to remove adapters and 13 nt from the 5′ end to mitigate bias in the random priming of library preparation. Reads were further cleaned to remove low quality base calls (<20) and short reads (<40 nt). Reads were mapped to the PGSC DM genome v 4.03 (Sharma *et al.* 2013) using the following parameters: mismatch cost = 2; insertion cost = 3; deletion cost = 3; length fraction = 0.9; similarity fraction = 0.8; max number of hits per read = 10. Expression values were reported in RPKM (reads per kilobase gene model per million mapped reads). Duplicate samples were used in an unpaired empirical analysis of differential gene expression and p-values were corrected for false discovery rate (FDR). Following this, the genes were filtered based on an FDR p-value <0.05 and an absolute value fold-change >2.0. This resulted in 78 annotated genes classified as differentially expressed.

### AN2 construct generation

All constructs were created using the pCambia 1305.1 vector as a backbone, excising the 35S promoter and GUS sequences by restriction digest (*Bam*HI and *Bst*EII) and replacing them with the relevant promoter/gene combination. Genomic sequences of the purple haplotype promoter and gene sequences were cloned from the inbred DRH_P_ 28-5. Since sequence data were not available for DRH_P_ 28-5 at the time, primers were designed to conserved regions within purple-flowered individuals of a sequenced monoploid panel (Hardigan *et al.* 2016). *AN2* cDNA was cloned directly from the DRH_P_ samples used for RNA-seq analysis (above). The following primers were used to engineer compatible restriction sites onto the promoter (*Bam*HI) and coding (*Bst*EII) sequence while a conserved *Xba*1 site located in the first exon was used to join promoters and coding sequences: An2pro2 (AATTAT**GgaTcc**TCTTGGTTTTTCTTTTCATATTTATAC), An2proXba1 (ACCAGC**TCTAGA**AGGAACAAGATGCC), An2CDSExon1 (TTGGGAGTGAGAAAAGGTTCATGG), and AN2CDSBstEII (ATATTA**ggtGacc**CCCTAGTACAAGTAGTAGTACAATACC). Verified products were ligated into the pJET 1.2 cloning vector and sequenced (Thermo Scientific CloneJET PCR Cloning Kit #K1232). pJET plasmids were digested with the appropriate enzyme (*Bam*HI and *Xba*I for the promoter; *Xba*I and *Bst*EII for the coding sequence) and the promoter was triple-ligated with the coding sequence into the pCambia 1305.1 binary vector. Binary vectors were introduced to ElectroMAX *Agrobacterium tumefaciens* LBA4404 cells (Invitrogen #18313-015). Primers AN2cDNAF (GTATCCCTAGTACAAGTAGT) and AN2cDNAR (ACAACATATCATGAATATTGCCA) were designed from cDNA of StFlANs and used to amplify genomic DNA extracted from *in vitro* leaf tissue of DRH 28-5; the two resulting bands were Sanger sequenced at the Virginia Biocomplexity Insitute Core Facility.

### Plant transformation

Plant transformation was carried out as described by Rooke and Lindsey (1998) with minor modifications. The resulting shoots were allowed to root in basal MS (Murashige and Skoog 1962) media (4.43 g l^-1^ MS salts, 3% sucrose, 7 g l^-1^ agar, pH 5.7-5.8). Once the rooted shoots were established, the media was washed off and plants transferred to peat pellets prior to placement in greenhouse ground-beds for phenotyping.

### Genomic analysis of the F locus

To determine the sequence of the white-colored allele of the *F* locus, we performed whole genome sequencing on the DRH_W_-derived monoploids as described previously (Hardigan *et al.* (2016). The purple allele of the *F* locus was obtained by cloning the gene from the homozygous DRH_P_ 28-5 inbred. Whole genome sequencing alignments were examined for variation at the *F* locus using Integrated Genome Viewer (Robinson *et al.* 2011). Sequence and phylogenetic analyses were performed using the Lasergene suite (DNASTAR, Inc., Madison, WI) and sequences of *StAN1 (*AGC31676) and *PhAN2 (*A4GRV2) were retrieved from the UniProt database (The Uniprot Consortium 2017). Identification of miniature transposable elements (MITEs) near the *AN2* locus was performed using RepeatMasker to query the representative potato MITE sequences retrieved from the P-MITE database (Chen *et al.* 2014) against the white-flowered DM reference genome v 4.04 (Potato Genome Sequencing Consortium 2011).

### Data availability

RNA-seq reads for the purple and white flower bulked samples are available in the National Center for Biotechnology Information (NCBI) Sequence Read Archive (SRA) under BioProject ID PRJNA636502. Whole genome shotgun reads of DM and the monoploids from Hardigan *et al.* (2016) are available under BioProject PRJNA287005. Whole genome shotgun reads of DRH_W_M (DMRH F1 monoploid sample DMRH 16-FL 2 D) is available in the NCBI SRA under BioProject PRJNA335820. Supplemental material available at figshare: https://doi.org/10.25387/g3.12869627.

## Results

### Genetic mapping of flower color in a diploid segregating population

**Figure 2:**
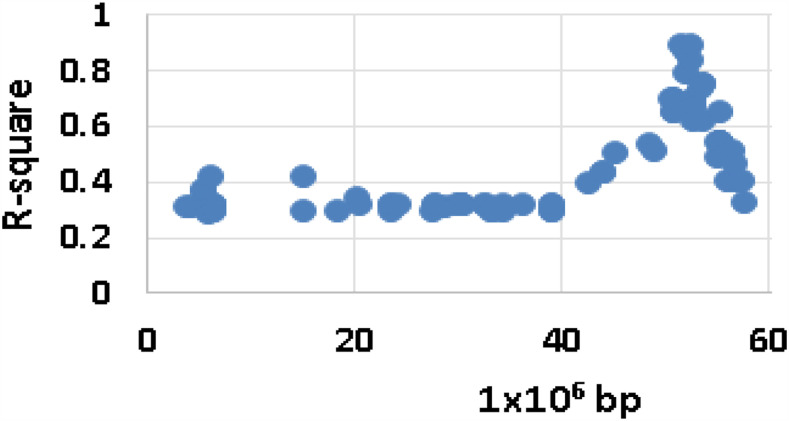
Significant SNPs linked to flower color in the DRH F_1_ segregating population are located on the distal end of chromosome 10. The x-axis is distance in base pairs along chromosome 10 and the y-axis is the R-square value from associations of SNPs with flower color state.

An F_1_ population segregating for flower color was derived from a cross between a white-flowered homozygous individual, DM, and a purple-flowered heterozygous individual, RH (Peterson *et al.* 2016). The F_1_ generation (DRH) displayed an approximate segregation ratio of 1:1 (51 purple DRH_P_: 44 white DRH_W_; χ^2^ = 0.38, *P* = 0.54), with all individuals being either white- or purple-flowered. The approximate 1:1 ratio and complete penetrance of the flower color phenotype indicated a single gene segregating from the heterozygous purple-flowered RH was the likely cause. Analysis of previously generated genotype data for this population (Peterson *et al.* 2016) identified SNPs significantly linked to the phenotype on the distal end of chromosome 10 (Figure 2). This finding is consistent with previous literature regarding the *F* locus for flower color (van Eck *et al.* 1993) which mapped the locus to the same approximate position using restriction fragment length polymorphism analysis.

### Transcriptomic analysis of purple *vs.* white flowers

To identify which genes were differentially expressed between white and purple flowers and potentially identify the transcriptional regulator for flower-color determination, we conducted RNA-seq using bulked RNA from pools of white and purple corollas from individual F_1_ lines. Using a cutoff of FDR-corrected p-value <0.05 and a minimum fold-change of two, we identified 78 genes that were significantly differentially expressed between the purple and white flower bulked samples (Supplementary Table 2). The gene with the greatest fold-change difference (500× greater in DRH_P_) was an R2R3 MYB transcription factor (PGSC0003DMG400019217) with 99% identity to a gene annotated by PGSC on Spud DB Genome Browser v4.03 as *AN2* on the distal end of chromosome 10 and 58% identity to *PhAN2* (Supplementary Figure 2). Henceforth, we will designate this gene as *StFlAN2* (*Solanum tuberosum Flower AN2*) to avoid confusion.

In addition to *StFlAN2*, 16 genes within the phenylpropanoid and anthocynanin biosynthetic pathways were differentially expressed between DRH_P_ and DRH_W_ bulked samples including bHLH-encoding *ANTHOCYANIN 1* (*StbHLH1*, initially called *StAN1*; (Zhang *et al.* 2009b; Payyavula *et al.* 2013), which was significantly up-regulated in DRH_P_ samples (Supplementary Table 2; Figure 1). Additionally, genes associated with fruit ripening were up-regulated in DRH_P_ samples, including four pectate lyase genes and three pectinesterase genes. Lastly, up-regulation of a subset of primary metabolism genes in the DRH_W_ samples was observed, including photosystem subunits, ribulose bisphosphate carboxylase/oxygenase, and fructose-1,6-bisphosphatase (Supplementary Table 2). These results indicate that the *StFlAN2* transcriptional regulator is a likely candidate gene to control the anthocyanin synthesis pathway involved in flower-color determination of potato.

### Transgenic recovery of the purple-flower phenotype

**Figure 3:**
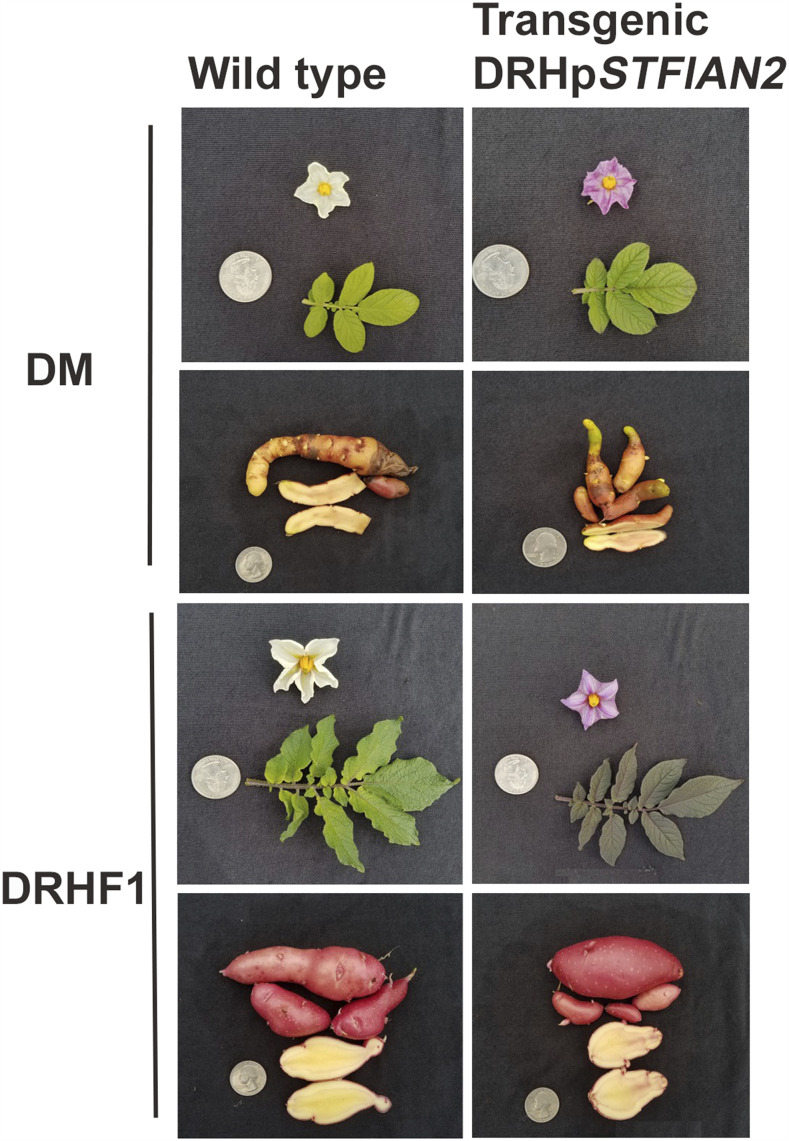
Exemplar phenotypes from transgenic complementation of DM and a white flowered DRH F_1_ (line number 171) with the promoter and cDNA of *StFlAN2* cloned from DRH_p_ 28-5, a purple flowered advanced inbred line fixed for purple flowers. The DM transgenic line shown is DM-DRH_p-ne_::cDNA-4 and the F_1_ line is DRH F_1-171_-DRH_p-ne_::cDNA-26.

To empirically test whether *StFlAN2* does confer the purple-flower phenotype, we cloned the *StFlAN2* promoter from genomic DNA and the coding sequence from cDNA of DRH_P_ 28-5, an inbred (S_5_) individual fixed for purple flowers. Interestingly, the promoter cloned from DRHp 28-5 is 1000 bp shorter than would be expected from the DM reference genome sequence and lacks the miniature transposable element (MITE) present in the DM promoter. When the *StFlAN2* construct was introduced into either the white-flowered DM or a white-flowered DRH F_1_ background, all of the resulting transgenic regenerants (seven and six independent transgenics for DM and F_1_, respectively) had purple flowers (Figure 3). These results suggest expression of *StFlAN2* is sufficient to convert plant lines with a white-flower phenotype to purple-flowered plants. In the particular DRH F_1_ transgenic line (DRH F_1_-_171_-DRH_p-ne_::cDNA-26) shown, possible pleiotropic effects on leaf pigmentation and tuber flesh can also be observed; however, this effect was not consistent among all the transgenic lines, indicating this phenomenon was possibly dependent on the transgene insertion site (Supplementary Figure 3).

### Characterization of the StFlAN2 locus

**Figure 4:**
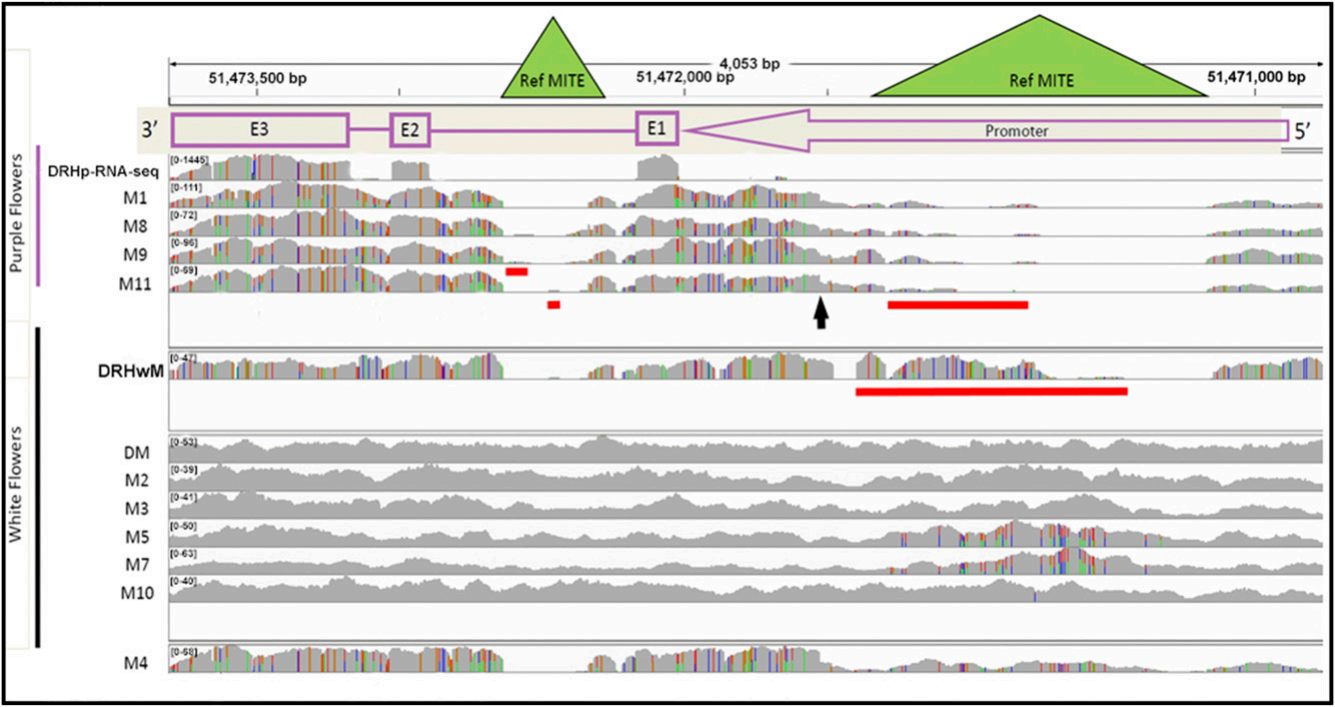
IGV image of sequencing coverage of the DRH F_1_ bulk segregant and monoploid haplotypes sorted by flower color aligned to chr10:51,474,000..51,469,500 of DM PGSC v4.03 Pseudomolecules. (Note that DM is white-flowered.) DRH_p_-RNA-seq represents the RNA-seq from the purple DRH bulk whereas all other tracks are whole genome sequencing. The gene structure *StFlAN2* is displayed with a purple arrow representing the promoter and boxes representing each exon (reading from right to left as the gene is on the bottom strand). Copy number variation is apparent in all purple tracks except for DRH_p_-RNA-seq (as only one paralog is expressed). This copy number variation manifests as multicolored bars in the allele frequency histogram which would otherwise be considered two separate SNP states in a heterozygous background. The locations of the two reference MITEs are displayed with green triangles. The black arrow highlights the start of reduced read depth from the 3′to the 5′ end of the promoter sequence in the purple monoploids. The red horizontal lines indicate regions of discontinuous alignment within MITE reads.

Having identified *StFlAN2* as the likely cause of differences in anthocyanin accumulation within the DRH population, we next performed in-depth sequence analysis of the locus in available purple- and white-flowered lines. The nucleotide sequence of the *StFlAN2* locus was analyzed by alignment in IGV (Figure 4) of RNA-seq data from the purple DRH bulk segregant pool (DRH_P_-RNA-seq) and whole genome shotgun sequence (WGS) data from (1) the white-flowered monoploid derived from a DRH_W_ individual (DRH_W_M); (2) the DM reference genome; (3) four purple-flowered monoploids (M1, M8, M9, M11); and (4) six white-flowered monoploids (M2, M3, M4, M5, M7, M10) reported previously (Hardigan *et al.* 2016). Two monoploids from Hardigan *et al.* (2016) were omitted; M6 because it never flowered and M12 because it was an interspecific hybrid with *Solanum chacoense*.

The locus and the regulation of *StFLAN2* expression appear to be more complex than initially expected. The bicolored lines in the histograms of the WGS alignments of purple-flowered monoploids (M1, M8, M9, and M11) in Figure 4 would ordinarily indicate heterozygosity; however, as these are monoploid plants with no possibility of heterozygosity, we interpret this as copy number variation (CNV) in the form of gene duplication of the *StFlAN2* locus. The duplication event appears to have encompassed only the 3′ end of the promoter, as evidenced by a drop in read depth (black arrow in Figure 4). Furthermore, both *StFlAN2* loci in the purple-flowered monoploids lack the MITEs found in the promoter region and second intron of the DM reference genome, as demonstrated by a lack of continuous reads aligned to the DM reference in this region (Figure 4). A small number of reads mapped to the MITE regions in these alignments, but exhibit lack of continuity against the DM reference on both 5′ and 3′ ends, indicating likely spurious read alignments (red lines in Figure 4). The alignment of DRHp-RNA-seq Sanger sequence to the reference genome revealed complete identity to DM in exon 1, two SNPs present in exon 2 and 18 SNPs plus two indels (6 bp) in exon 3 (Supplementary Figure 4 – note that the gene reads from right to left). All 18 SNPs in the third exon are present in all of the read alignments of the purple flowered bulk RNA sample (Figure 4 and Supplementary Figure 4). We interpret this lack of apparent CNV in the DRH_P_-RNA-seq sample to indicate that only one of the two *StFlAN2* copies in DRH_P_ and the purple-flowered monoploids is expressed. We performed a sequence search of the haplotype-resolved RH genome assembly (http://solanaceae.plantbiology.msu.edu/index.shtml) using Sanger sequences derived from amplification of DRH 28-5 genomic DNA using primers designed to DRH_p_ cDNA sequences. *StFlAN2* is located on haplotype 1 of chromosome 10 in RH and has an additional two paralogs within ∼100 kbp, confirming our prediction of CNV in DRH 28-5. In contrast, only one allele of *StFlAN2* or its paralogs was detected in the corresponding region of haplotype 2 of chromosome 10 of RH. Specifically, the one that differs from the DM reference genome and is derived from the duplication event (Figure 4). To distinguish between the two copies, we have named the non-expressed copy found in all lines *StFlAN2ne* and the duplicated copy found in purple-flowered lines *StFlAN2e*.

DRH_W_M, although different from the DM reference, shows no CNV, indicating it harbors only one copy of the *StFlAN2* locus. In addition, it lacks both portions of the promoter MITE and the intronic MITE; the alignments (red line in Figure 4) in the central region of the promoter MITE in DRH_W_M exhibit lack of continuity against the DM reference on both 5′ and 3′ ends, indicating that this region may exhibit spurious read alignments (Figure 4). Part of the region downstream of the promoter MITE position that has reduced read depth in the purple-flowered monoploids is lacking in the DRH_W_M promoter.

With the notable exception of M4, the remaining white-flowered monoploids (DM, M2, M3, M5, M7, and M10) appear to have only a single copy of the *StFlAN2* locus (Figure 4). The apparent CNV encompassing the MITE in the promoter region of M5 and M7 indicates the assignment of a duplicate MITE to this region. This also explains the increased relative read depth of this region for M5 and M7 (Figure 4).

The white-flowered monoploid M4 is an exception. Like the purple-flowered monoploids, it also appears to have a duplication of the *StFlAN2* locus. Both copies lack the intronic MITE, as demonstrated by a lack of reads mapped to the DM reference in this region (Figure 4). However, based on read-depth variation, it appears that only one of the copies contains the promoter MITE (Figure 4). The M4 promoter MITE matches the duplicated MITE in M5 and M7, whereas the remaining part of the promoter matches the promoters of the purple monoploids. Based on the white flower color, neither copy is expressed.

**Figure 5:**
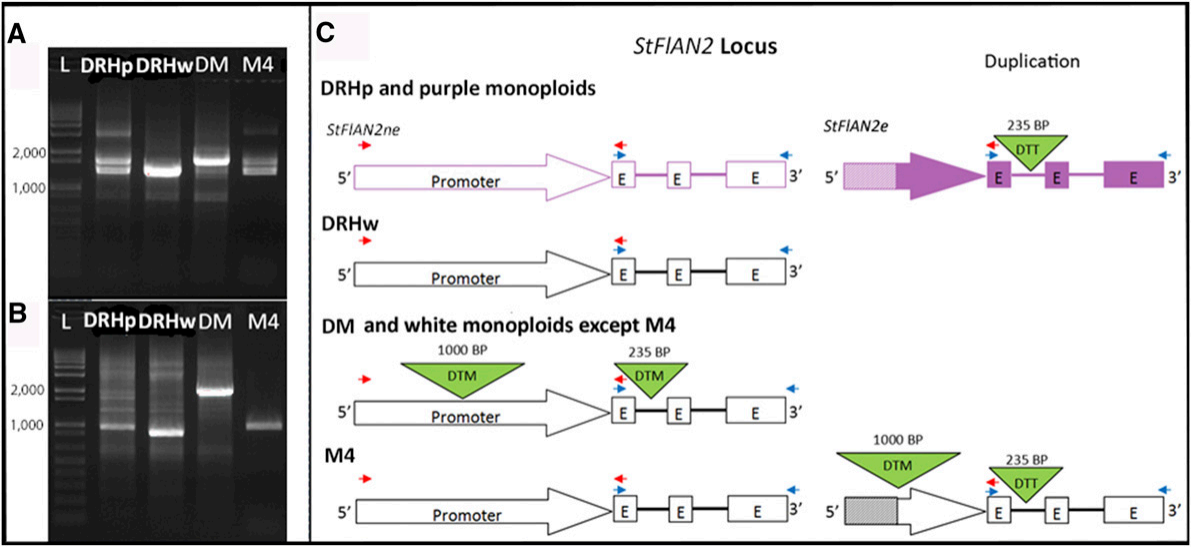
A) PCR amplification of genic sequences of *StFlAN2* haplotypes: DRHp (from DRHp 28-5), DRHw (from DRHwM), DM and M4; B) PCR amplification of promoter sequences of *StFlAN2* haplotypes as in A. In both A and B, L denotes Invitrogen 1kb+ ladder; C) Scheme of the *StFlAN2* locus in DRH_P_, DRH_w_, DM, and a white flowered monoploid haplotype (M4). DRH_P_ includes both the non-expressed (*StFlAN2ne*) and expressed (*StFlAN2e*) paralogs. Arrows represent promoters while boxes and lines represent exons (E) and introns, respectively. Green triangles depict MITEs inserted into either promoter or intronic sequences labeled by superfamily. Excluding transposons, shapes are filled to indicate expression whereas unfilled shapes indicate lack of expression. Striped fill indicates unknown sequence which lacks homology to the reference genome. Locations for primers used in cloning and PCR are displayed with red (promoter) and blue (CDS) arrows. Note: Differences in haplotype ideograms in C are reflected in band size and number in A and B.

We exploited the primers used to clone the cDNA from DRH_P_ to amplify and analyze the genic *StFlAN2* sequence from the genomes of DRH_P_, DRH_W_, DM, and M4 (Figure 5A). The results for DRH_P_ indicate that the genic region of the expressed copy (*StFlAN2e*, whose exons match the cDNA) also contains a MITE. However, although it is of the same size (235 bp), it is of a different superfamily (Tc1/Mariner; DTT) than the MITE found in the DM reference *StFlAN2* promoter and intron (Mutator; DTM). Hence, the whole genome shotgun sequencing results from the purple-flowered monoploids (M1, M8, M9, and M11) do not show alignments to the DM reference intronic DTM MITE (Figure 4). The genic region of the non-expressed copy (*StFlAN2ne*, that matches the DM reference genome) does not contain any MITE and is consequently shorter, leading to two different-sized PCR amplicons between 1 and 2 kb (Figure 5A). Amplification of the DRH_W_ *StFlAN2* genic region yielded an amplicon identical to the shorter of the two found in DRH_P_, matching the results of the whole genome shotgun sequence data for this line (Figure 4). Amplification of the DM *StFlAN2* genic region yielded an amplicon of similar size as the longer of the two found in DRH_P_, but with a DTM MITE in the first intron, matching the DM reference genome (Figure 4). Lastly, amplification of the M4 *StFlAN2* genic region yielded two amplicons identical to the two found in DRH_P_, one that matches the DM reference genome (*StFlAN2ne*) but lacks the DTM MITE, and one whose exons match the DRH_P_ cDNA (*StFlAN2e*) and contains a DTT MITE in the first intron (Figure 5A).

We next exploited the primers used to clone the promoter from DRH_P_ to amplify and analyze the *StFlAN2* promoter sequence from the genomes of DRH_P_ (from DRH_P_ 28-5), DRH_W_ (from DRH_W_M), DM, and M4 (Figure 5B). Only a single band was obtained for each promoter amplification, as the forward primer was designed based on the DM reference genome and only matched the single copy (*StFlAN2ne*), the reverse primer is at the start of the first exon (Figure 5C). An approximately 1 kb amplicon was obtained from DRH_P_, matching the DM reference genome minus the DTM MITE (Figure 5B). The DRH_W_ promoter amplicon was slightly smaller, matching expectation based on the whole genome shotgun sequencing analysis (Figure 4) that indicated it lacked the promoter MITE and was missing 20 bp downstream of the promoter MITE position. The amplicon obtained from DM was approximately 2 kb, representing the promoter including the DTM MITE. Lastly, the amplicon obtained from M4 was identical to the DRH_P_ amplicon, representing the promoter without the DTM MITE and indicating that the MITE reads from the shotgun sequencing analysis (Figure 4) must have come from the duplicated locus.

These results lead us to the following interpretation of how *StFlAN2* expression varies in the different white- and purple-flowered lines (Figure 5C). The purple-flowered DRH_P_ and monoploids (M1, M8, M9, and M11) harbor two copies of the gene; *StFlAN2ne*, which is not expressed and contains neither a promoter MITE or an intronic MITE, and *StFlAN2e*, which is expressed and contains an intronic DTT MITE. The white-flowered DRH_W_ harbors only one copy, identical to DRH_P_ *StFlAN2ne*, which is not expressed and contains neither a promoter MITE nor an intronic MITE. The white-flowered DM and monoploids (M2, M3, M5, M7, and M10) also harbor only one copy, which is not expressed but contains both a promoter DTM MITE and an intronic DTM MITE. The white-flowered monoploid M4 harbors two copies of the gene; *StFlAN2ne*, which is not expressed and contains neither a promoter MITE or an intronic MITE, and StFlAN2e*, which is also not expressed and, similar to the purple-flowered DRH_P_ and monoploids, contains an intronic DTT MITE but, in contrast to the purple-flowered DRH_P_ and monoploids, also contains a DTM MITE in its promoter. Lastly, the transgenically complemented, purple-flowered DM line with the inserted *StFlAN2* construct harbors an additional gene copy with the *StFlAN2ne* promoter from DRH_P_ driving the *StFlAN2e* cDNA, indicating that this promoter can be active outside of the *StFlAN2* locus.

## Discussion

### StFlAN2 underlies the F locus for flower color in potato

With a combination of genetic mapping, RNA-seq, transgenic complementation, and genomic sequence analysis, we show that a *PhAN2* homolog, *StFlAN2*, is the regulator of floral anthocyanin production in potato flowers, at least in the populations studied. This matches *PhAN2* function in petunia, where it is also responsible for anthocyanin production in flowers. The 1:1 segregation pattern for flower color in the DRH F_1_ segregating population combined with a presence/absence phenotype imply the flower color is due to segregation of a single regulatory gene. If there had been a continuum of color or a more complex segregation pattern, a biosynthetic gene might have been a more likely cause, as Śliwka *et al.* (2017) hypothesized in their study on flower color intensity. Genetic mapping revealed a significant QTL on the distal end of chromosome 10 which, when combined with previous reports that this region harbors the requisite *F* locus for flower color (van Eck *et al.* 1993), provides further support for this assertion. RNA-seq analysis shows *StFlAN2* to be the most differentially regulated gene between purple- and white-flowered genotypes. Finally, the shift from white to purple flower color in the DM background by a transgenic construct provides yet another level of support that *StFlAN2* indeed controls expression of anthocyanin biosynthetic genes in potato corollas.

### Regulatory effects exerted by StFlAN2

Transcriptome analyses provides insight into which genes are affected by the expression cascade initiated by *StFlAN2*. These genes span the gamut of anthocyanin biosynthesis, starting with the initial flux of carbon from aromatic amino acids into the phenylpropanoid pathway catalyzed by phenylalanine ammonia lyase (PAL), to the first committed step of the flavonoid pathway, chalcone synthase (CHS), and finally ending with the assortment of enzymes involved in anthocyanin structural modification, such as glucosyl-, acyl-, and glutathione transferases (Fraser and Chapple 2011). Within the gene annotation of the potato genome (DM 1-3 516 R44 v3.04), there are 11 *PAL* genes, although only six of them appear to be full length copies (Supplementary Table 3) and two of them appear to be partially duplicated. One of the two PAL genes identified in our study, PGSC0003DMG400031365, is highly expressed in purple corollas of RH compared to white-flowered DM [Univeristy of Toronto BAR ePlant website (http://bar.utoronto.ca/eplant_potato/) accessed 7/24/2020). The other, PGSC0003DMG400023458, has little or no expression in DM and high expression in the stems of RH. With regard to *CHS* genes, there are 11 annotated in the potato genome (DM 1-3 516 R44 v3.04), most of which appear to be full-length (Supplementary Table 3). Four of the 11 are highly expressed in the purple flowers of RH (http://bar.utoronto.ca/eplant_potato/) accessed 7/24/2020) and two are expressed in the white flowers of DM, including the gene identified in our study (PGSC0003DMG400019110) which appears to be expressed in flowers regardless of color. Similar to what is observed for the tuber-specific *StAN1* and *StAN2* [also referred to as *StMYBA1* (Liu *et al.* 2016)] MYBs (Payyavula *et al.* 2013), this study finds that expression of *StFlAN2* correlates with the expression of genes within the phenylpropanoid pathway that do not contribute to production of anthocyanins, such as P-coumaroyl quinate/shikimate 3′-hydroxylase and caffeoyl-CoA O-methyltransferase. As these genes are believed to contribute to the production of other phenylpropanoid-derived compounds, their increased expression is more likely attributable to an increased flux of precursors than the direct action of *StFlAN2* itself (Vanholme *et al.* 2010; Fraser and Chapple 2011).

The remaining differentially expressed genes are not involved directly in the production of anthocyanins but are possibly attributable to the physiological consequences of anthocyanin production. Three major functions stood out among these genes: up-regulation of genes involved in cell-wall degradation and ripening, down-regulation of polyphenoloxidase genes, and down-regulation of genes involved in photosynthesis and carbon fixation in purple corollas. Although care was taken to sample only recently opened, turgid flowers for all samples, the abundance of up-regulated pectinesterase genes and pectate lyase genes in the purple corollas suggests that they were biochemically further along in the ‘ripening’ process than white corollas. The more than 20-fold reduction in polyphenoloxidase expression relates to anthocyanin levels, as these enzymes are known to mediate anthocyanin degradation (Pifferi and Cultrera 1974; Jiang 2000). The light-shielding function of anthocyanins could serve as an explanation for lower expression of photosynthetic genes, such as RuBisCo and photosystem subunits, in purple corollas. Anthocyanins have been shown to protect against photoinhibition by absorbance of light and limiting permeation into the leaf (Steyn *et al.* 2002).

### Structural variation of the StFlAN2 locus

The analysis of the floral *StFlAN2* locus in DM, DRH, and its inbred derivatives as well as a white-flowered monoploid indicates a dynamic local genetic terrain, with both transposon activity and copy number variation. Jung *et al.* (2009) identified a tuber-specific *PhAN2* homolog as the regulator of anthocyanin production in tuber skin. Subsequent analysis showed that the region harboring *StAN1*, located approximately 300 kb distal to the *StFlAN2* locus described here, to be replete with MYB homologs, including multiple pseudogenes and at least one other functional gene, named *StAN2* [also called *StMYBA1* (Liu *et al.* 2016)], which is responsive to cold and drought stress and responsible for anthocyanin accumulation throughout the plant (André *et al.* 2009; D’Amelia *et al.* 2014; D’Amelia *et al.* 2018). Hence, the region – and the MYB homologs by extension – has been affected by multiple duplications leading to subfunctionalization in which no less than three separate potato genes have been referred to as *AN2* due to their shared homology with *PhAN2* (Supplementary Table 4); for clarity we will continue to refer to the floral locus as *StFlAN2*.

We report here that another duplication, giving rise to the paralog *StFlAN2e*, is responsible for segregation of corolla anthocyanin production in the DRH population. In tomato (*Solanum lycopersicum*), there is also a duplication of *PhAN2* homologs on chromosome 10; both homologs are functional but not redundant, with only one regulating fruit color (Kiferle *et al.* 2015). It has been observed that the regions harboring *PhAN2* homologs in *Petunia inflata* and *P. axillaris* are also remarkably dynamic, with little synteny between the two despite the recent divergence of the species, perhaps due to high transposon density (Bombarely *et al.* 2016). Thus, it is possible that complexities surrounding the *StAN1* and *StFlAN2* loci are the current manifestations of a region especially prone to structural variation for many of the Solanaceae due to a heightened density of repetitive elements and lack of pleiotropic effects of the *PhAN2* homologs themselves (Bombarely *et al.* 2016).

Interestingly, the promoter of non-expressed *StFlAN2* allele (*StFlAN2ne*) that is present in both the DRH_P_ and DRH_W_ F_1_ segregants is active when it is inserted elsewhere in the genome, as all 13 independently regenerated transgenic lines derived from two different genotypes generated for this study had purple flowers (Figure 3). Ectopic expression in leaf and stem tissue was apparent in some of the transgenics. This suggests that there are likely some repressive *cis*-elements nearby but outside of the region cloned here and/or a repressed chromatin state. Sequence organization of the *StAN1* promoter has also been suggested to be important for expression of anthocyanin synthesis in potato leaves and tuber skin (Strygina *et al.* 2019). This may also explain why the duplicated paralog *StFlAN2e* that is only present in the DHR_P_ F_1_ segregants and the DHR_P_ 28-5 inbred line is expressed and confers purple flower color, if the duplication removed it from the influence of this putative repressive *cis*-element.
